# Supplementary material for: Development and validation of predictive models for diabetic retinopathy using machine learning
Source: PLoS One. 2025 Feb 24;20(2):e0318226. doi: 10.1371/journal.pone.0318226 (PMC11849896; doi:10.1371/journal.pone.0318226)
Supplement: Attachment — Submitted filename: Response to Reviewers.docx [file pone.0318226.s003.docx]

**Dear Reviewers,**

**Thank you for providing the opportunity to revise our manuscript, titled “Development and validation of predictive models for diabetic retinopathy using machine learning”. We greatly appreciate the time and effort invested by the reviewers to improve the quality of our work. Below, we provide our point-by-point response to each of the reviewer’s comments.**

****Reviewer #1****

1. ****"The authors have presented a comprehensive comparison approach, however, the authors fail to present more recent publications that support their comparative analysis. More recent publications should be incorporated too."****

**To address this, we have incorporated more recent publications into the discussion and comparative analysis section. These include studies published in 2022–2024 that explore the application of machine learning models in diabetic retinopathy prediction and validate our findings. Relevant citations have been added to strengthen our analysis.**

2. ****"There are multiple Typo and grammatical mistakes which need to be corrected by the authors. Such as in line 283, there are two full stops."****

**We have thoroughly reviewed the manuscript to identify and correct all typographical and grammatical errors, including the redundant punctuation at line 283.**

3. ****"The use of citations for the dataset used is not consistent with the citation style used in the rest of the document."****

**We have corrected the formatting of the dataset citation to ensure consistency with the citation style used throughout the manuscript.**

4. ****"The quality of figures is bad and in some cases not readable. Also, for some reason, there is Fig2.pdf icon present at line 193. Kindly look into this issue."****

**We apologize for the oversight regarding the quality of the figures and the misplaced Fig2.pdf icon. The figures have been updated to high-resolution versions, and the icon has been removed. Additionally, figure legends have been revised for clarity.**

5. ****"Though the contents of the tables have been discussed, the figures provided have not. Kindly provide an extensive discussion regarding the result achieved for each figure."****

**Thank you for pointing this out. We have added an in-depth discussion of the results depicted in each figure, highlighting their relevance to the study’s findings and implications. The updated discussion is included in the results section.**

****Reviewer #2****

1. ****"The paper is very well written in an organized manner."****

**We thank the reviewer for their positive feedback and are glad that the manuscript's organization was appreciated.**

2. ****"The paper fails to provide a comprehensive review and comparison with existing published work on Diabetic Retinopathy."****

**We have addressed this by expanding the literature review and integrating a broader comparison with existing studies on diabetic retinopathy. The revised section now includes more detailed discussions on prior work and highlights how our findings align with or differ from these studies.**

3. ****"Recommended with minor addition."****

**We have incorporated the suggested additions and revisions based on the feedback provided, ensuring the paper is comprehensive and addresses all the reviewers' concerns.**

**We sincerely hope that the revised manuscript meets the reviewers' expectations. Thank you again for the opportunity to improve our work. Please let us know if further modifications are required.**

**Sincerely,**

**Bin Yang**
